# Couplelinks - an online intervention for young women with breast cancer and their male partners: study protocol for a randomized controlled trial

**DOI:** 10.1186/s13063-014-0534-8

**Published:** 2015-01-29

**Authors:** Karen Fergus, Saunia Ahmad, Deborah L McLeod, Joanne Stephen, Sandra Gardner, Amanda Pereira, Ellen Warner, Wendy Carter

**Affiliations:** 1 Department of Psychology, Faculty of Health, York University, 4700 Keele Street, Toronto, Ontario M3J 1P3 Canada; 2 Patient and Family Support Program, Odette Cancer Centre, Sunnybrook Health Sciences Centre, 2075 Bayview Avenue, Toronto, Ontario M4N 3M5 Canada; 3 Cancer Program, Queen Elizabeth II Health Sciences Centre, Victoria 11-006, 1276 South Park Street, Halifax, Nova Scotia B3H 2Y9 Canada; 4 Department of Oncology, Faculty of Medicine, Cumming School of Medicine, Health Sciences Centre, University of Calgary, 3330 Hospital Drive NW, Calgary, Alberta T2N 4N1 Canada; 5 Ontario HIV Treatment Network, 1300 Yonge Street, Toronto, Ontario M4T 1X3 Canada; 6 Dalla Lana School of Public Health, University of Toronto, 155 College Street, Toronto, Ontario M5T 3M7 Canada; 7 Division of Medical Oncology, Odette Cancer Centre, Sunnybrook Health Sciences Centre, 2075 Bayview Avenue, Toronto, Ontario M4N 3M5 Canada; 8 Dr. Wendy Carter, 7 Admiral Road, Coach House, Toronto, Ontario M5R 2L4 Canada

**Keywords:** Cancer, Breast, Couple, Online intervention, Internet, Psychosocial, Protocol, Randomized control trial

## Abstract

**Background:**

Young breast cancer survivors (aged 50 years and under) and their partners are at an elevated risk for relationship distress and poor psychological adjustment relative to older age couples. Limited availability of time and resources and the distance to travel are major barriers to engaging in evidence-based psychosocial support programs. This paper describes the study protocol of a novel, manualized online intervention called Couplelinks that was developed to improve relationship adjustment and psychological wellbeing of young couples affected by breast cancer. Couplelinks is a custom-designed website offering a professionally facilitated, couple-centered intervention that entails informational, experiential, and interactive components.

**Methods/Design:**

A total of 80 heterosexual couples from across Canada in which the female partner has been diagnosed with a primary breast cancer will be recruited and randomized to a treatment or waitlist control group. Six dyadic learning modules form the core of the program and will be undertaken on a weekly basis. The manualized online intervention involves psycho-education and experiential exercises to enhance communication, coping ability, mutual empathy, and perspective-taking in relation to cancer. An online facilitator who is a trained mental health professional will guide and support couples throughout the process. Data collection will occur at baseline, at post-treatment or eight weeks into the waiting period, and at the three-month follow-up assessment. Primary outcome measures include the Revised Dyadic Adjustment Survey (RDAS) and Dyadic Coping Inventory (DCI) scores, and secondary outcome measures include the Hospital Anxiety and Depression Survey (HADS) score.

**Discussion:**

Couplelinks is one of the first internet-based psychological interventions to improve the psychosocial adjustment of couples coping with a life-threatening illness such as cancer. If successful, the design of this program as described in this paper makes a valuable contribution to the literature on the delivery of couple-focused psychosocial interventions, both within and outside of oncology.

**Trial registration:**

This trial was registered with ClinicalTrials.gov (identifier: NCT01089764) on 17 March 2010.

## Background

Women aged 50 years and under account for only 18% of newly diagnosed breast cancer (BC) cases [1], yet they experience significantly greater levels of cancer-related distress and poorer quality of life relative to women diagnosed later in life [2-7]. Population-based age cohort studies have found that younger women are at greater risk for experiencing functional declines in physical, social, and psychological domains for as long as 10 years post-cancer treatment [2,8,9]. Younger women also suffer significantly greater levels of depression, anxiety, and fears that the cancer will recur [10-12]. Chemotherapy-induced menopause among younger women is related to greater sexual difficulties and physical symptoms than if menopause occurred prior to cancer diagnosis and treatment [11].

Such age-related discrepancies in BC patients’ adjustment to the illness are in part related to the abrupt and developmentally premature physical and social changes associated with the diagnosis and treatment of cancer that most older women have either already come to terms with, or for whom such changes are no longer relevant. These unique challenges may include: the loss of fertility and therefore a loss of the choice to conceive children [2,4,11,13,14]; not having children because of the fear that pregnancy-related hormonal changes may provoke a cancer recurrence [15]; premature menopause with its associated hot flashes, and sleep and mood disturbances [2]; concerns about their ability to adequately care for, and address, the needs of their young children, and how to communicate with them about the illness in a way that is appropriate to the child’s developmental stage [16,17]; interruptions to early-stage careers [4,18]; and/or relationship distress [3,19]. Financial strains as a result of taking time off work for treatment or out-of-pocket expenses for childcare and/or housekeeping, and the increase in expenses due to treatment are particularly problematic for younger couples whose full income potential may not yet be realized [20]. Moreover, social comparison processes with similarly aged peers increase the salience of these losses for younger women for whom the illness is ‘off-time’ developmentally [21,22].

Although the quality of general social support helps mitigate much of younger BC patients’ distress [23,24], the support from an intimate partner is particularly important to women’s adjustment to BC [25]. Stressors that couples commonly contend with include: renegotiation of family roles and responsibilities, for instance, the well partner may take on additional responsibilities in various areas such as childcare [26]; feelings of inequity, for instance, caregivers may feel as though they are taking on a disproportionately greater amount of work in the relationship, or the patient may experience guilt over not being able to contribute to the relationship as before [27]; reduced sexual engagement and social activity [2,28]; increased financial strain due to out-of-pocket expenses or lost wages of the ill partner [29]; difficulty communicating about fears and distress related to cancer [25,30]; preoccupation with thoughts of mortality [12], and for partners in particular, thoughts of loss and abandonment [31,32]. Such challenges may be even more pronounced for younger couples. For example, declines in sexual engagement may be intensified as a result of premature menopause and related loss of libido, vaginal dryness, and painful intercourse [33]. Younger couples often have to come to terms with the loss of goals and dreams that older couples may have already realized, such as starting or growing one’s family [21,34]. Moreover, younger couples’ relationships are likely less resilient to stressors such as BC as older couples, relative to younger couples, are more likely to have a better understanding of each other, and by virtue of having already experienced hardships together, are more likely to have established collaborative coping skills [35].

Spouses’ coping behaviors and ratings of marital support, communication, and/or relationship satisfaction correlate significantly with BC patient’s psychosocial outcomes [36-39]. Partner behaviors that have been found to contribute positively to patient adjustment include attentiveness and responsiveness to the patient partner’s emotions, while at the same time being able to share one’s own feelings about the cancer [25,30,40-42]. On the other hand, withdrawal, minimization, criticism, and advice-giving on the part of the spouse negatively affects patient coping [43,44]. BC patients often report that their spouses tend to be instrumentally supportive rather than emotionally [45,46], and male partners themselves report a lack of knowledge and skills in being able to respond constructively to their ill partners’ complex needs [47,48]. Male partners also report experiencing distress in their role as caregivers characterized by: feelings of guilt, depression, and inadequacy [23,24,47]; concerns over increased role strain and multiple demands for their time [26]; lack of adequate knowledge in supporting their children [49]; anxiety about sexual intimacy, including having negative reactions to the partner’s mastectomy site [50]; and lastly, fear of recurrence of the cancer after active treatment [31,32].

### Rationale for online couple based intervention

Given the wealth of research highlighting the interdependency of partners’ distress and coping on their subsequent adjustment to cancer, psychosocial interventions that are couple-focused have been recommended (for examples, see [3,51,52]), particularly for younger couples who are more vulnerable to distress and poorer quality of life [3,17,33]. While conventional couple interventions have demonstrated efficacy in reducing distress for couples coping with breast cancer [53-57], they may not be a feasible option for younger couples who are already living very busy lives with additional demands, such as caring for young children and balancing domestic duties and active careers alongside attending medical appointments. Indeed, studies have found that the primary reasons breast cancer patients and their partners do not participate in or complete couple-focused psychosocial interventions are limited time, distance, and lack of willingness to travel [57,58]. Furthermore, traditional counselling approaches may not adequately address the unique and complex needs of younger BC couples.

The present paper describes an innovative, online psycho-educational intervention currently under investigation that addresses the void in the psychosocial support available to younger couples facing a breast cancer diagnosis. This intervention, which is tailored specifically to this group’s unique needs, is delivered through a custom-designed website called ‘Couplelinks.ca’. Couplelinks provides a professionally facilitated, couple-centered intervention that includes informational, experiential, and interactive components. Given the numerous barriers to younger couples’ engagement in psychosocial services, an online relationship enhancement intervention that is both accessible and flexibly delivered offers a compelling alternative to conventional counselling.

Most online psychosocial interventions currently available for breast cancer patients are support groups that interact via virtual discussion boards [59-63]. Participation in internet-based support groups has been associated with reductions in social isolation, depression, and cancer-related trauma, and increased feelings of personal empowerment and self-esteem [62,64-70]. Such outcomes have, for example, been demonstrated in a Canada-wide initiative, ‘CancerChatCanada’, to offer professionally facilitated group support to individuals affected by cancer through a secure, online platform [71]. Each group employs real-time (synchronous), text-based communication as the medium for group interaction, and is structured like a face-to-face support group, with approximately eight group members and a group leader [72]. Asynchronous groups using bulletin board technology that are moderated by a mental health professional have similarly shown beneficial effects for cancer patients (for examples, see [59,70]).

The Comprehensive Health Enhancement Support System (CHESS) is another example of a successful online support program for people affected by cancer. The service was originally developed for breast cancer patients, but has more recently been studied with other populations such as the medically underserved and/or economically disadvantaged sub-groups of BC patients [73,74], and caregivers to lung cancer patients [75]. CHESS provides an online platform where women with BC have access to health monitoring and decision-making tools, and can ask questions of health professionals as well as provide and receive emotional and informational support from other BC survivors [75]. Such programs provide BC survivors with cancer-specific education to help with treatment-related decisions, as well as managing physical and psychological symptoms. The site also offers opportunities for social bonding with others facing similar experiences.

Apart from greater access to professional support, the online modality allows for flexibility in terms of time, and has the added benefit of overcoming various other barriers to accessing psychosocial support, such as childcare and transportation issues [61,62,64,76,77]. Studies have found that younger women with breast cancer demonstrate a strong preference for receiving psychosocial support via online programs [78], and that men are more inclined to open up about their feelings and discuss sensitive information in relation to cancer in an online versus face-to-face context [60]. Younger men in particular have shown reluctance to make use of conventional forms of psychosocial support (such as counselling services) as it may conflict with their self-concept and notions of strength and self-reliance [79-81].

Taken together, these findings suggest that the internet would be a very promising modality through which to offer support to younger women with breast cancer and their partners. Couplelinks aims to build on the success of previous online support programs for individuals with cancer by: (1) targeting couples (versus individuals), (2) addressing the specific psychological and informational needs of younger women with BC and their partners, and (3) combining didactic learning with experiential learning on topics relevant to coping with BC as a younger couple.

### Hypotheses

We hypothesize that women and their male partners who participate in the Couplelinks program will demonstrate significant improvement on measures of relationship adjustment and satisfaction, dyadic coping, and intimacy, and measures of psychological distress and adjustment, as compared to participants in the control condition.

## Methods/Design

### Intervention development and content

#### Conceptual framework of the online program

The structure and content of Couplelinks was derived based on theory and empirical evidence that couples who construe the illness as a shared problem (not something ‘belonging’ to the ill partner) are better able to engage in mutually supportive interactions that promote their adjustment [82-85]. Enhancing partners’ sense of ‘we-ness’ in relation to breast cancer as a means for alleviating distress is grounded theoretically in the work of Reid *et al*. and Fergus *et al*. [82,83,86-88]. Their framework for understanding and treating distressed relationships integrates family systems and constructivist theory pertaining to how partners make meaning, and how such meaning-making processes influence their interpersonal dynamic. Within this framework, the more partners construe themselves as being part of their relationship, also referred to as couple identity or we-ness, the greater their overall relationship resilience [83,84,87]. Adopting a we-oriented approach to coping (also known as ‘we-coping’ [88-90] or ‘dyadic coping’ [91]) accomplishes several things. Firstly, it mitigates the potential for either partner to feel isolated and alone in his or her coping. It also de-centers the couple and the greater relationship from the illness, countering the tendency for the relationship to become defined by the losses and stressors associated with BC. Lastly, in creating an adaptive separation between the couple and the illness, the couple is afforded the distance and greater perspective necessary to discuss the illness constructively and problem-solve around it [92].

When partners adopt a ‘we’ attitude in relation to cancer (seeing it as ‘our’ problem rather than hers alone) their individual coping efforts are coordinated with each other, allowing them to better manage the stress related to the illness. Both partners are likely to express their needs to the other, and be able to request and receive support from their partner, even if each does so differently [88]. High levels of we-ness among couples dealing with BC have been found to contribute to greater relationship adjustment, increased closeness between partners, and lower levels of individual distress [88]. Assuming a we attitude and functioning as a unit in relation to cancer has consistently been associated with adaptive outcomes for couples [88,89,93,94]. Not only does taking a we approach to the cancer enhance adjustment, but successfully coping with cancer itself can further contribute to strengthening the relationship and create greater closeness [47,95].

Other models informing the development and design of the program include: (1) Rolland’s [85] developmental model of couple adaptation to illness and specifically, the disruptions experienced by younger couples when age appropriate goals and milestones (such as having a child) are impeded because of the illness and the long-term consequences of treatment (such as premature menopause and infertility); and (2) Gottman’s [96] well-validated theory of healthy relationship functioning and interaction patterns that promote relationship adjustment, such as having a higher ratio of positive-to-negative interactions (and correspondingly, positive-to-negative affect) in the relationship, or having an accurate understanding of one’s partner’s thoughts and feelings, and broader goals, dreams, and preferences.

Theoretical integration of these frameworks supports the overall objectives of the Couplelinks intervention, which are to: (1) enhance couple’s sense of we-ness as a means to better enabling partners to manage stressors associated with BC; and (2) facilitate partners’ making meaning of the experience of BC in a way that draws them closer together, despite the emotional hardship, losses, and grief experienced due to the illness. These goals are achieved by progressively guiding the couple through exercises designed to foster empathy, perspective-taking, constructive communication and listening skills, emotional and physical intimacy, and positive affect in the relationship. By foregrounding we-ness relative to the illness, the couple is in a better position to externalize BC and experience themselves as an ‘us’ in relation to ‘it’. While the couple engages in exercises intended to support one another within the relationship, BC is integrated into each learning module as both a current stressor whose impact on each partner needs to be accurately understood and acknowledged, and a topic or problem to be collectively addressed.

### Phase I: pilot research

The content and design of Couplelinks is entirely original and was developed by members of the research team in consultation with an advisory committee comprised of couples with a history of BC and stakeholders in the BC community. The content and functionality was developed to address the needs and concerns specific to younger breast cancer couples. Couplelinks was designed to be informative, engaging, and interactive. In the phase I trial, the intervention was pilot-tested using a non-randomized, pre-post test design to assess whether couples found the website easy to navigate and use, the website content to be clear and coherent as well as informative about couples coping with BC, and the program convenient, flexible, and enjoyable (as intended) [97]. Qualitative and quantitative analyses of measures assessing treatment satisfaction and perceived benefits from 10 couples that completed phase I suggested that the aforementioned objectives were being met. The majority of participants reported that they found the program easy to use, convenient, and beneficial in that it provided a safe and structured opportunity to discuss difficult topics in relation to cancer that, for many participants, had been avoided or simply not addressed prior to the program. Feedback from phase I trial couples on the website functionality and program informed improvements to the intervention and website. The improved, second iteration of the intervention is being tested in a phase III randomized controlled trial (RCT) currently underway across Canada and is described in this paper.

### Intervention protocol

Six experiential, dyadic learning modules (DLMs) that form the core of the program (with an optional seventh module) are undertaken by the couple on a weekly basis in consultation with a Couplelinks facilitator.

#### Dyadic learning modules

Each module is designed to teach a different relationship principle. The basic structure of every DLM is as follows: (1) description of a basic relationship skill with examples; (2) exercises partners are asked to engage in so that they can learn the skills experientially; and (3) discussion between partners of what they learned followed by logging reflections and feedback about the exercise (see Table 1 for a description of each module and its objectives). The exercise and feedback components involve logging thoughts, observations, and reflections online that provide both the interactive learning for the couple, as well as information for the facilitator to respond to. For instance, module three, ‘Creating Connection’, focuses on helping partners increase positive interactions relative to negative interactions and is based on marital research that includes the work of Gottman [96] which found that healthier relationships tend to demonstrate a greater ratio of positive to negative interactions. In module three, the difference between supportive and unsupportive behaviors and their impact on relationship quality is explained, and then partners are asked to observe and log during the week their partner’s ‘bids’ for support and to observe and describe their own ‘turning towards’ and ‘turning away’ behaviors from their partner. All modules from phase I were retained in phase III with the exception of module five from phase I, a listening skills module, that was changed to an optional seventh module in phase III based on feedback from the pilot study. Module five has been replaced by a module that focuses on rebuilding physical intimacy, a change made in response to feedback from couples in the pilot phase looking for help with restoring the sexual wellbeing of their relationship, which is often negatively affected by cancer treatment and the stress of the illness in general.

**Table 1:** **Dyadic learning modules**

**DLM**	**Theme**	**Goal**	**Activity**
1	Celebrating our Strengths	To create an opportunity for partners to reflect upon and communicate about their individual and shared strengths. Individual strengths consist of qualities in the other that one values, enjoys, or admires. Shared strengths include those that define the couple relationship as strong or resilient and help in the process of coping with BC.	Independently, each partner enters 10 positive qualities about their partner online. Then the partners brainstorm together about the strengths they share as a couple in general and log these online. They are then asked to together choose from this list the couple strengths that they bring to bear on their experience with BC. Their entries are transformed into an image of a tree with their individual strengths listed in the roots and their couple strengths listed in the foliage. The couple is asked to review the image and discuss these together.
2	Understanding your Partner’s Inner World	To help partners more accurately understand the other’s thoughts and feelings in relation to BC based on the assumption that previous relationship schemas may have to be revised or altered in the context of the illness.	Independently, each partner answers a series of questions about their own and their partner’s preferences and experiences progressing from trivial to more serious topics (including cancer-related). These lists are then reviewed together in order to stimulate discussion and clarification.
3	Creating Connection	To help partners become more aware of the other person’s ‘bids’ for interaction and support, and to pay attention to their own ‘turning toward’ and ‘turning away’ behaviors on a day-to-day basis [96].	Over the course of the week, each partner is asked to attend to his or her own turning toward and away behaviors as well as his/her partners turning toward behavior . These are tracked and recorded online. At week’s end, the couple reviews and discusses their entries that appear in chart format.
4	Facing Cancer as a Unified Front	To assist couples in adopting a team orientation in relation to BC (a sense of ‘us’ versus ‘it’). Also, to foster the attitude that the illness is a shared experience (not ‘belonging’ to woman with cancer).	Couples are guided through an exercise designed to get them thinking metaphorically about cancer, and then to create a visual representation of the illness in order to fortify sense of ‘we-ness’ in relation to cancer.
5	Getting Physical	To assist couples in reconnecting physically and sensually as a stepping stone to re-engaging sexually, as many couples find their sexual life is disrupted by treatment.	Independently, each partner recalls and records a physically pleasurable shared time from their past. The couple then discusses each memory. Next, couples engage in a Sensate Focus exercise.
6	Looking Back and Moving Forward	To assist couple with moving forward after BC by situating the illness in the context of the larger relationship history and by having the couple consider new goals and directions for themselves (particularly in the wake of lost goals and dreams).	Together partners co-construct a relationship timeline illustrating pivotal events and/or periods in their shared history (high and low points). The website transforms relationship events and phases inputted by the couple into a relationship timeline. This relationship timeline forms a basis for discussion.
(optional)	Intentional Dialogue	To learn a communication skill that partners can use to share their concerns more effectively and increase their understanding of each other’s perspective.	Couples are taught active listening skills by watching an instructional video clip of another couple demonstrating an Intentional Dialogue. Couples are asked to then attempt this skill on their own. First, using a neutral topic and then using a more meaningful topic. Completion dates are entered online.

Adapted from Fergus et al., 2014 [97].

Couples are asked to undertake each module on a weekly basis and adhere to a six to eight-week timeframe. This timeline is reasonable based on our pilot work where we found that couples took approximately eight weeks on average to complete the six modules after taking into consideration vacations or unexpected interruptions. The modules are consecutive and cumulative with subsequent modules building upon knowledge and skills acquired through previous ones. Couples only gain access to the next module once the previous module has been completed. Access to subsequent modules is granted by the facilitator who first reviews and provides feedback to the couples as described below.

##### The Couplelinks facilitator

Each couple in the treatment group is linked with a facilitator with whom they regularly interact as they proceed through the modules. Interactions with the facilitator predominantly occur through the Couplelinks website ‘Dialogue Room’ (DR). The DR is an asynchronous (facilitator and couple are not online simultaneously) communication platform that utilizes bulletin board technology to enable each partner to correspond directly with their facilitator, as well as with one another, as they progress through the program. Additionally, the facilitator has two pre-arranged phone ‘check-ins’ after module two and four. Each time a couple completes a module their facilitator is notified by an automatically generated email from the website. After reviewing each partner’s online reflections and feedback about the module, the facilitator provides the couple with feedback via the DR in order to validate their insights and new learning, acknowledge any challenges or difficulties they may have experienced, and explain the objectives of the subsequent module and, where applicable, how it builds on previous themes arising for the couple. At this point the facilitator grants the couple access to the next module. Couples also use the DR, for example, to ask questions about the program, to update the facilitator on their progress, or to inform the facilitator of any events that will conflict with an upcoming due date. The facilitator is also available for additional telephone consultations as necessary; however, the pilot study showed such consultations to be rare and, when undertaken, usually done to resolve minor technical issues.

There will be a total of five mental health professionals with experience in psycho-oncology with a range of disciplinary backgrounds (psychology, nursing, and social work) serving as Couplelinks facilitators. The current facilitators have been instrumental in developing this novel form of support and/or are well versed in the principles and techniques of providing Couplelinks facilitation as detailed in the Couplelinks program facilitation manual [98]. In addition to studying the manual, newer members of the facilitation team receive individualized coaching and instruction on how to offer the intervention through ‘role play’ with a trainer (a clinician member of the research team), who acts as a ‘dummy’ couple, progressing through each DLM and interacting online with the training facilitator as an actual couple would.

The program facilitation manual describes in depth the guiding principles for online facilitation for the program overall, and for each of the seven DLMs. It also provides numerous examples of text-based feedback to each module, including facilitator responses to various challenges that may occur, such as a couple whose participation is interrupted due to investigation of a new, concerning symptom. All facilitators are individually supervised by either the first or second author (KF and SA) which includes an ongoing review of their interactions with active couples as logged and visible in the back-end of the Couplelinks interface (described below). All facilitators also attend regular peer supervision teleconference meetings led by the first and second author. Together, both forms of supervision ensure treatment fidelity; that facilitators adhere closely to the principles of online facilitation as articulated in the Couplelinks manual and maintain consistency in their method of facilitation. Which of the five facilitators a couple is assigned to will depend on whether or not the facilitator has space in his or her caseload, the facilitator’s discipline, and the corresponding regulating body’s stance on inter-jurisdictional practices. For instance, a clinical counsellor licensed in British Columbia may serve couples from other provinces, while a psychologist licensed to practice in Ontario cannot facilitate couples who reside outside of his or her geographic jurisdiction.

### Front-end user interface

Every detail of the website, from its ‘look and feel’ to the content, was conceived with an eye toward being inclusive of both genders, clear and easy to follow, and engaging and enjoyable. The homepage includes an introductory video introducing the facilitator. Additionally, each DLM has its own corresponding home page and a video interview of a couple from the phase I trial of Couplelinks sharing their thoughts and reflections on that specific DLM. Another important consideration in the design of the intervention was that it be convenient and adaptable to the demanding schedules of younger couples. Pilot testing indicated that each module takes, on average, one hour in total to complete. In order to assist with planning and time management, an automated scheduler called the ‘Lesson Tracker’ that lists each module, its constituent steps, and estimated time for completion was introduced in the phase III version of the website and located on the homepage. Each time one step of a module is completed it registers automatically in the Lesson Tracker. Partners’ work is saved and they can log back in at a later time and use the Lesson Tracker to locate where they left off. Several optional readings are available on the Couplelinks website that focus on concerns most relevant to younger couples dealing with BC, and relational coping including fertility after BC, coping with premature menopause, sexuality and intimacy during and after treatment, parenting through breast cancer, and living with uncertainty and fears for the future. As not all topics pertain to each couple, there are no required readings.

### Back-end administrative interface

There is an administrative back-end to Couplelinks with different levels of access for each member of the Couplelinks team. The facilitators have access to the module data for the couples they are directly working with, and a ‘contact notes’ section to communicate with the research team overseeing the project. The facilitator uses the administrative interface to review a given couple’s progress on modules and their feedback, as well as to advance the couple to the next module. Additionally, facilitators can log contact notes to describe all interactions that occur outside the Couplelinks website, such as the phone call check-ins or emails that may have been exchanged due to difficulty with the website. The research coordinator ((RC) co-author, AP) and two clinician scientists (KF and SA) who are in charge of overseeing timely completion of modules and supervising facilitators have access to all outcome measure responses, facilitator contact notes, and all entries logged by all participants as they progress through each DLM.

### Confidentiality and security

The website is password protected; users must be authenticated in order to access the website. Additionally, all transmission of content and online interactions are encrypted. All data from the website are backed up on secure servers.

### Phase III: study design

This is a multicenter, national prospective two-arm RCT of Couplelinks. Approximately 80 eligible couples will be randomized to either the treatment condition or a waitlist control condition, using randomized block design with a block size of 2 (1:1), stratified by province of residence. The randomization list is generated using PROC FACTEX in SAS (Statistical Analysis System) version 9.3, SAS Institute, Cary, USA.

This study was approved by the Research Ethics Boards of all participating hospitals and cancer centers, namely Sunnybrook Health Sciences Centre (reference number: 300–2009), York University (reference number: 2010-119), Cancer Care Manitoba - Research Resource Impact Committee (reference number: 2013–017), University of Manitoba (reference number: H2013:119), University of British Columbia - British Columbia Cancer Agency Research Ethics Boards (reference number: H10-00300), and Queen Elizabeth II Health Sciences Centre - Capital Health Research Ethics Board (reference number: CDHA-RS/2010-357).

### Inclusion and exclusion criteria

The RC completes an eligibility screen with interested participants. Eligible couples are those in which the woman has received a diagnosis within the last 36 months of invasive breast carcinoma (non-metastatic), or ductal carcinoma *in-situ*, at or before the age of 50 years. While we did not have specific criteria for stage of cancer treatment, we inform potential participants when they contact the RC for information about the project that it is probably best to begin at or nearing the end of active treatment. Feedback from phase I suggested that participants are more inclined to participate at this stage, and this finding is supported by evidence indicating that couples are often more vulnerable to experiencing conflict at this time due to differing expectations around recovery from BC [47]. Couples must be in a committed, heterosexual relationship (for example, married, cohabitating, engaged, or steadily dating for at least six months at the time of participation). Both partners must fluently read and write in English and have access to a computer with a reliable internet connection.

Couples are excluded from the study at the eligibility screening stage if: (1) either partner self-reports current suicidal ideation and/or attempts, or a diagnosis of a serious and currently active mental illness such as psychotic disorders, bipolar disorder, depression, or substance abuse that is not being successfully managed with psychiatric treatment, and therefore may potentially interfere with his or her capacity to benefit from the program; (2) the couple is either currently in couple counselling or expresses the intent to undertake counselling during the five-month study period; (3) one or both partners self-report interpersonal violence. If it becomes evident at any point over the course of the couple’s involvement in the program that their relationship distress is so significant that they would not benefit from the program, or their participation is significantly exacerbating rather than mitigating their distress, they are asked to discontinue the project and are referred to a face-to-face couple’s counsellor.

### Recruitment

Eligible patients from across Canada and their partners are being identified and informed about the study by their health care providers at collaborating institutions in Ontario, Nova Scotia, Manitoba, and British Columbia. In our pilot study, we found that active forms of recruitment enhanced enrolment. Patients who have been approached by their health care provider and express an interest in learning more about the program are asked for consent to have their contact information forwarded to the RC, who then follows up with the potential participant by phone to provide her (or him) with information about the program. Additionally, flyers are displayed across various hospitals and cancer agencies. Announcements have been made at hospital meetings, BC support groups, and community cancer related workshops for young women. The advertisement is posted on websites commonly accessed by young women with BC, social media, and the Couplelinks informational webpage that is linked to these.

### Procedure

Once a potential participant (typically the patient rather than the well partner) connects with the RC, she is informed about the nature of the project. This potential participant is asked to discuss the project with her partner and, if both are interested, the RC proceeds with scheduling a phone interview with each partner separately to confirm whether they both meet eligibility criteria. When screening couples, in addition to the inclusion and exclusion criteria previously described, the RC assesses partners’ access to, and comfort with, the internet and whether they are likely to be able to keep up with the weekly time commitment for the program. Partners who anticipate interruptions (for instance due to travel) for a duration longer than two weeks are asked to delay enrolment until after they return. To avoid the confounding effects of other interventions, couples are informed of the expectation that they refrain from participating in couple-related psychosocial interventions until they have completed the five-month study term.

If both participants meet the eligibility criteria, the RC proceeds with the informed consent process over the phone, which includes reviewing the objectives of the study, requirements of the participants, how their information will remain confidential, and participants’ right to withdraw at any time. Any concerns or questions are addressed and if the couple is satisfied, they are told they will be mailed a hard copy of the consent form that we request they review, sign, and return and that they may contact the RC again if they have any further questions. Once the signed informed consent form is received, the couples are randomized to the waitlist or intervention condition.

Next, each partner is separately emailed a unique personal login ID and password which they use to complete all questionnaires, starting with the baseline questionnaires. The couples in the treatment group complete additional questionnaires as described in the measurement section below. Participants are informed that their responses to the surveys will not be shared with their partner, and they are instructed to complete the surveys without consulting one another. Once the time one (T1) measures are completed, the RC informs participants of their group assignment. Participants assigned to the treatment group are informed of the name of their Couplelinks facilitator. The facilitator schedules a phone meeting with the couple to introduce her or himself, clarify expectations of participants, answer questions, and review important information about the website and the intervention. The facilitator then sends the couple their login instructions via email.

Both groups of participants complete standardized questionnaires online at T1, time two ((T2) at post-treatment for treatment and for waitlist groups, taking place eight weeks after T1), and time three ((T3) three months after completion of T2 measures). Participants in the treatment group are also asked to complete a post-treatment interview at T2. Couples in the waitlist group are given the option of receiving the intervention once they have completed the T3 questionnaires; if they choose to participate in the intervention, their completed T3 questionnaires will be used as their pre-treatment (baseline) measurement, and they will be asked to complete surveys at post-treatment and one last time 3 months after posttreatment.

### Measurement

**Figure 1:**
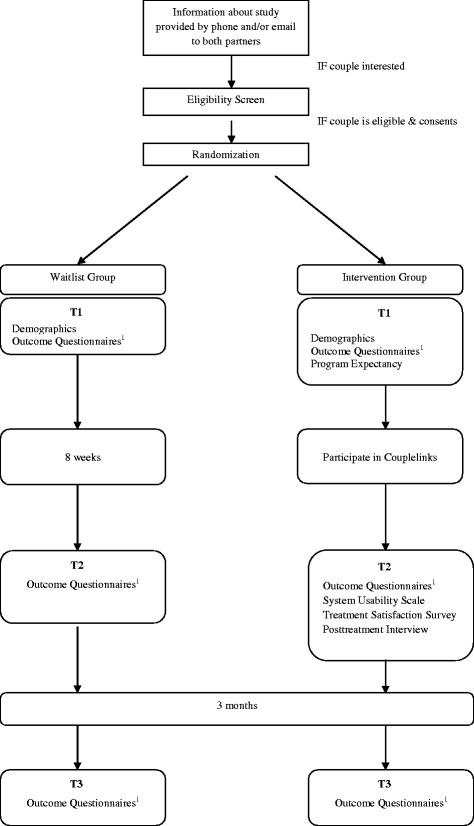
**Flowchart of a randomized controlled trial (RCT) of Couplelinks.** ^1^Outcome Questionnaires include: BCRM = Breast Cancer and Relationship Measure, DCI = Dyadic Coping Inventory, FACT-B = Functional Assessment of Cancer Treatment - Breast (female only), HADS = Hospital Anxiety and Depression Scale, KMSS = Kansas Marital Satisfaction Survey, MMQ = Maudsley Marital Questionnaire, RDAS = Revised Dyadic Adjustment.

The primary outcome of this study is improvement in relationship adjustment and dyadic coping. Secondary outcomes include improvement in mood (for example, anxiety or depression) and interpersonal communication. Self-reported socio-demographic variables are collected at T1 including: gender, age, ethnicity, education, employment status, relationship status, living arrangement, and number and gender of children. Self-reported disease and treatment information includes cancer stage, treatments received, status of treatment, and other health conditions. The assessment of the intervention’s effectiveness incorporates the effects of attrition and non-compliance into the analyses. Satisfaction with the intervention is assessed using a self-report questionnaire designed specifically for this study, and feedback for further improvements is obtained using a phone interview completed post-treatment. See Figure 1 for a flowchart of the trial with time points and corresponding measures.

The Revised Dyadic Adjustment Survey (RDAS) [99] is an abbreviated version of the longer and widely used Dyadic Adjustment Survey (DAS) [100], with comparable levels of reliability and validity. This 14-item measure assesses dyadic consensus, cohesion, and satisfaction. The Kansas Marital Satisfaction Scale (KMSS) [101] is one of the most commonly used global measures of marital satisfaction. It is comprised of three items on a seven-point Likert scale, asking respondents to identify their level of satisfaction with their partner, their marriage, and their relationship with their partner. Both the RDAS and the KMSS have demonstrated reliability and validity, and empirically derived cut-offs to differentiate between samples of couples being seen for couple intervention versus a community sample. The marital adjustment subscale of the Maudsley Marital Questionnaire (MMQ) [102] evaluates an individual’s satisfaction with his or her committed or married relationship. It has 10-items, each rated on a nine-point scale.

The Hospital Anxiety and Depression Scale (HADS) [103] is a 14-item four-point Likert scale measure that includes two subscales of seven items each, assessing anxiety and depression. It is a simple yet reliable and valid tool to assess the presence of a mood disorder in medical settings. Although developed for a hospital-based population, it has also been found to be a valid screening instrument for depression and anxiety in the general population [104]. The Functional Assessment of Cancer Therapy-Breast (FACT-B) - Physical Well-Being (PWB) [105] subscale is part of the widely used FACT-B that assesses quality of life among breast cancer patients. The PWB, a seven-item five-point Likert scale measuring physical functioning, is completed by the female partner only. This variable will be tested as a potential covariate. The Breast Cancer and Relationship Measure (BCRM) was developed by the study authors for this protocol to assess the degree to which partners experience breast cancer to have negatively affected their relationship. The scale has 10 items which participants rate on a scale from 0 (not at all) to nine (very much) depending on the degree to which each statement applies to them. An example item is ‘We are able to discuss difficult or sensitive issues related to breast cancer’.

The Dyadic Coping Inventory (DCI) [106] is a 37-item questionnaire that measures levels of stress-related communication and levels of supportive and negative dyadic coping behaviors when one or both partners are stressed, as well as perception of one’s partner’s stress-related communication and support. The scale consists of seven subscales, with four measuring self and partner stress communication, and supportive, negative, and joint dyadic coping. Additionally, two items capture the quality of the self-perceived level of overall dyadic coping. The Credibility Expectancy Questionnaire (CEQ) [107] is a six-item survey that assesses the participants’ expectations about how beneficial they believe the treatment will be, and how reasonable it is in their view that the treatment will address their problem. The questionnaire consists of two parts to evaluate such expectations and perceptions, with one part asking participants to answer the questions based on their rational thought process, and the other section in which they are asked to answer based on their emotions. The original measure assessed changes in trauma symptoms. The wording of the measure in this study refers to changes in relationship adjustment. This measure is administered to the treatment group at T1 only.

The System Usability Scale (SUS) [108] is a 10-item measure designed to capture the participant’s subjective, global perspective of the website’s usability, including the website complexity and the perceived need for support or training. This measure is administered to the intervention group at T2 only. The Treatment Satisfaction Survey (TSS) measure was developed specifically for this study and assesses the satisfaction of participants in the intervention group with the program overall and specific aspects of it (for example convenience and facilitator role). In addition to items requiring quantitative ratings, open-ended questions are asked regarding the general experience, the program’s perceived benefits, and areas for improvement. The purpose of this measure is to obtain feedback regarding different facets of Couplelinks, in order to inform program development and determine participants’ perceptions regarding the effective and less effective aspects of program experience. This measure is administered once at T2 to the intervention group. After completing the T2 questionnaires, a semi-structured interview designed specifically for this study is conducted over the phone with couples in the intervention group to get more detailed feedback about the intervention. Both partners are interviewed together and the interview takes 40-60 minutes.

### Sample size estimation and data analyses plan

All scale and subscale means and standard deviations will be computed and summarized. The primary hypotheses to be tested are that the degree of change or improvement between T1 and T2 on the RDAS, KMSS, MMQ, and DCI summary scales will be greater for the treatment group relative to the waitlist group. Given that data are non-independent (measurements are of individuals that belong to the same dyad and multiple measures are taken per individual across time) the hypotheses will be evaluated using multilevel models (MLM) [109]. MLM will allow for testing correlations between partners’ outcomes and whether there are differences in treatment effect for the patient versus her partner. The models will also be used to evaluate the degree of sustained change from T2 to T3, and whether treatment effects vary by severity of disease, age, and/or length of relationship, as well as aspects of the intervention such as level of engagement. MLM will also allow us to test whether there are any significant variations in treatment effect between facilitators. The impact of missing data will also be evaluated.

The sample size needed was based upon psychometrics of our primary outcome measure, the RDAS. Assuming a two-sided test, a standard deviation for mean change in RDAS from T1 to T2 equal to 3.4 (standard deviation of RDAS = 7.0, with a correlation of 0.9 between T1 and T2), an intracluster correlation of 0.3, an effect size of 0.5 for mean change of RDAS (difference in group means = 1.8), 80% power, and an alpha of 0.05, 36 couples will be required for each group (PASS, NCSS, Kaysville, USA, cluster-randomization power analysis) [110]. The total sample size was calculated as 36 × 2 × 2 = 144. The sample size will be increased to 160 (40 couples per group) due to an expected attrition of 10% of the participants across time, based on previous research in couples and BC [111]. In addition to the questionnaires, qualitative data will be collected from the open-ended evaluation of each DLM following its completion.

## Discussion

The potential for Couplelinks to fill a void in the support options available to both younger women with BC and their male partners is great. The intervention, if proven effective, will be the first empirically validated online support tool for heterosexual couples with the female partner belonging to the highly distressed and inadequately served population of young BC survivors. Moreover, this validated program will address the consistently demonstrated need for targeted resources that appeal to their male partners. If effective, this standardized psychosocial intervention can be readily incorporated into existing psychosocial oncology programs, with the added benefit of independence from geographic disparities. Moreover, as one of the first couple interventions of its kind, Couplelinks will help set a clinical and scholarly precedent for the delivery of online support to couples in distress. The ultimate impact of the study will be the creation of an accessible, empirically validated tool that could help to improve the quality of life of future young couples coping with BC, regardless of geographic location.

Assuming effectiveness is demonstrated, the next step would be to refine the treatment and training manual, and disseminate the program to psychosocial oncology professionals. They, in turn, would have access to the website on a cost-recovery basis in order to offer the program within their own communities and provinces. Furthermore, as an empirically validated prototype, Couplelinks may be adapted for other subgroups of BC survivors and their partners, such as same-sex or middle-aged couples, or for couples dealing with other types of cancer.

## Trial status

Recruitment for this project has been underway since April 2011. The estimated date of completion is June 2015.

## Abbreviations

BC: Breast Cancer
BCRM: Breast Cancer and Relationship Measure
CEQ: Credibility Expectancy Questionnaire
CHESS: Comprehensive Health Enhancement Support System
DCI: Dyadic Coping Inventory
DLM: Dyadic Learning Modules
DR: Dialogue Room
FACT-B: Functional Assessment of Cancer Treatment
HADS: Hospital Anxiety and Depression Survey
KMSS: Kansas Marital Satisfaction Survey
MLM: Multilevel Models
MMQ: Maudsley Marital Questionnaire
RCT: Randomized Controlled Trial
RC: Research Coordinator
RDAS: Revised Dyadic Adjustment Survey
SUS: System Usability Scale
T1: Time One
T2: Time Two
T3: Time Three
TSS: Treatment Satisfaction Survey.
